# Survey data of intra-household decision making and smallholder agricultural production in Northern Uganda and Southern Tanzania

**DOI:** 10.1016/j.dib.2017.07.040

**Published:** 2017-07-25

**Authors:** Chris M. Mwungu, Caroline Mwongera, Kelvin M. Shikuku, Fridah N. Nyakundi, Jennifer Twyman, Leigh Ann Winowiecki, Edidah L. Ampaire, Mariola Acosta, Peter Läderach

**Affiliations:** a International Centre for Tropical Agriculture (CIAT), Nairobi, Kenya; b International Centre for Tropical Agriculture (CIAT), Cali, Colombia; c International Centre for Tropical Agriculture (CIAT), Hanoi, Vietnam; d International Institute of Tropical Agriculture (IITA), Kampala, Uganda; e World Agroforestry Centre (ICRAF), Nairobi, Kenya

**Keywords:** Intra-household, Gender survey, Decision making, Agricultural production

## Abstract

This article provides a description of intra-household survey data that were collected in Uganda and Tanzania in 2014 and 2015, respectively. The surveys were implemented using a structured questionnaire administered among 585 households in Uganda and 608 in Tanzania. Information on decision making processes in agricultural production was collected from the principal adult male and female decision-makers in each household. The survey consisted of two parts. Firstly, the decision-makers, both male and female of each household were jointly interviewed. Secondly, individual interviews were carried out, questioning the decision-makers separately. The datasets include both household and individual level data containing numeric, categorical and string variables. The datasets have been shared publicly on the Harvard dataverse.

**Specifications Table**

**Table:** 

Subject area	Agricultural sciences
More specific subject area	Decision making and agricultural production in smallholder farms
Type of data	Categorical, string and numeric variables
How data was acquired	Intra-household surveys through face to face interviews were conducted using a structured questionnaire.
Data format	STATA (dta) files and CSV files in raw format.
Experimental factors	
Experimental features	
Data source location	Kilolo and Mbarali districts in the Southern Agricultural Growth Corridor of Tanzania (SAGCOT) and Nwoya district in the Acholi sub-region in Northern Uganda
Data accessibility	The data accompanying this article can be found online at**:**https://dataverse.harvard.edu/dataset.xhtml?persistentId=doi:10.7910/DVN/0ZEXKC

**Value of the data**

• The datasets can be used to compare how adoption of different Climate Smart Agricultural (CSA) practices and decision making affect agricultural production in rural farm households.
• The dataset provides a significant contribution on capturing important intra-household dynamics, often ignored in household data collection efforts. The following data were disaggregated by gender: plot-level farm production, land tenure, intra-household decision-making processes, access to agro-climate information services, perceptions of climate change, intra-household differences of awareness and adoption of various climate adaptation practices, and personal values.
• The key variables from rural households for both male and female respondents can be used for gender-disaggregated analysis of impacts of climatic risks on the welfare of rural agricultural households.
• Datasets are valuable in examining socio-economic, institutional and cultural factors influencing adoption of CSA practices among smallholder farmers, and to conduct cross-site comparisons.

## 1 Data

**Fig. 1:**
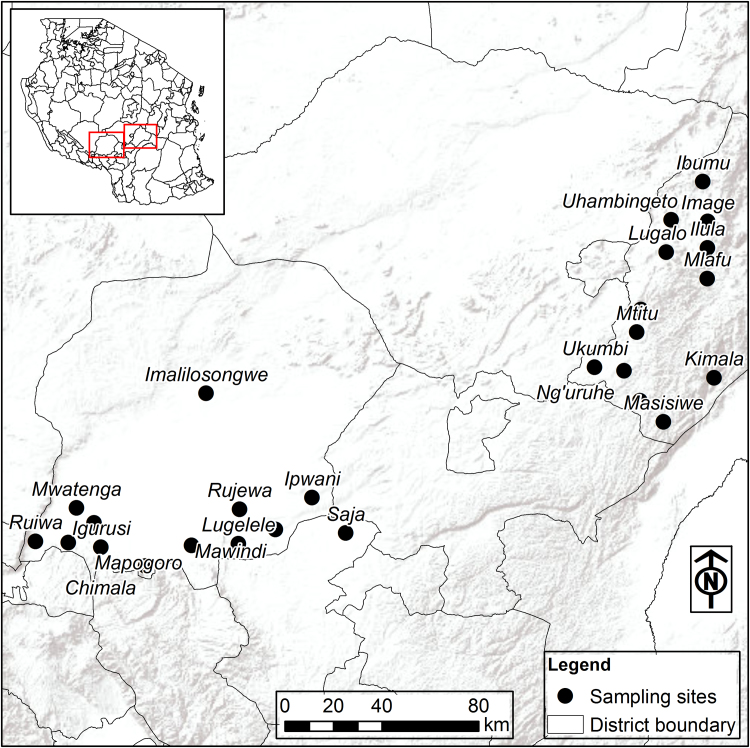
Map showing wards in Southern Tanzania where households were sampled.

**Fig. 2:**
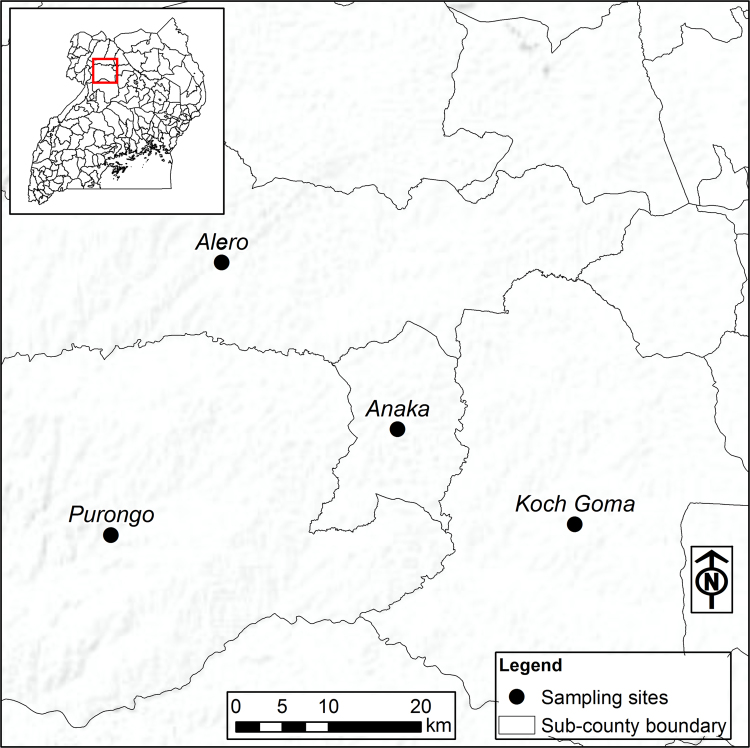
Map showing sub counties of Nwoya District in Northern Uganda where households were sampled.

The datasets described in this article were collected in Northern Uganda (Nwoya District) in 2014 and in the southern agricultural growth corridor of Tanzania (SAGCOT) in 2015. These data were collected by the International Center for Tropical Agriculture (CIAT), in collaboration with the International Institute of Tropical Agriculture (IITA). Both datasets comprise information about household characteristics, crop production and marketing activities, land ownership and tenure, intra-household decision making, asset ownership, access to credit and information, exposure, sensitivity, response to climatic stresses, personal values and farmers’ attitudes, and agricultural practices. The information on decision making, group membership, access to information, adoption of agricultural practices, and perceptions on climate change and adaptation were obtained by interviewing the principal adult male and female decision makers in each household separately. In addition to the data, a codebook describing value labels for variables and the questionnaires that were used for data collection are also available. However, identification variables such as farmer's names, GPS coordinates, village names are only available upon request (Fig. 1, Fig. 2).

## 2. Research design, materials and methods

Following [1], a stratified random sampling technique was used to sample 585 and 608 farmers in Uganda and Tanzania, respectively. In Tanzania, the first stage of sampling involved purposive selection of two districts namely, Mbarali and Kilolo. The two districts were chosen because they lie within the SAGCOT, a region largely targeted by the government of Tanzania and international donors for large agricultural investments [2], [3], [4]. The second stage of sampling involved generating a complete list of all wards and randomly selecting 50% to participate in the study, using a random number generator in Microsoft Excel. Using this criteria, 10 and 11 wards were selected in Mbarali and Kilolo, respectively. To arrive at the number of villages to sample in each ward, we based this on the desired total sample for the ward: 1) If less than 20 households were required we selected 1 village: 2) between 20 and 40 households, 2 villages were randomly selected; 3) and where total number of households required in the ward was above, 3 villages were selected randomly. We ended up with a total of 19 villages in Mbarali and 21 in Kilolo. The names of all the households in each of the selected villages was obtained, and then the desired sample were randomly selected. The Tanzania dataset was collected through Computer Assisted Personal Interviews (CAPI) using a pre-designed CS-Entry template.

In Uganda, Nwoya district was prioritized as it is a target district for a restoration of livelihoods programme financed by the Government of Uganda and the International Fund for Agricultural Development (IFAD) [2], [3], [5]. All the four sub-counties in Nwoya district namely, Anaka, Purongo, Koch Goma, and Alero were included. Since some sub-counties were bigger than others, a probability-proportionate-to-size (PPS) sampling technique was used. Within each sub-county, parishes were randomly selected, and villages were selected from parishes using PPS. Households were randomly selected from villages. The ultimate sample size comprised 585 farming households. The Uganda dataset was collected using paper questionnaires. Data were entered and verified in CSPro before being exported in STATA 14.1 and CSV formats. Both datasets were cleaned using Stata version 14.1 software to check for outliers and missing observations.

## Funding sources

Funding for this work was with support from the International Fund for Agriculture Development (IFAD) Grant number 2000000176.

## Supplementary material

Supplementary data and other materials accompanying this article are within this article and can also be found online at**:** https://dataverse.harvard.edu/dataset.xhtml?persistentId=doi:10.7910/DVN/0ZEXKC.
