# Supplementary material for: Survey data of intra-household decision making and smallholder agricultural production in Northern Uganda and Southern Tanzania
Source: Data Brief. 2017 Jul 25;14:302–6. doi: 10.1016/j.dib.2017.07.040 (PMC5544471; doi:10.1016/j.dib.2017.07.040)
Supplement: Supplementary material [file mmc4.pdf]

Household ID [ ] Male Respondent ID [ ] Female Respondent ID [ ]

General Information

Date (dd/mm/yyyy)	
Name of Household Head	
Name of Respondent	
Name of enumerator	
Name of second numerator	
Time the interview started (am/pm)	
Time the interview ended (am/pm)	
Name of Supervisor	
**Geographical location (provided by the site coordinator)**	
Country	
Region	
District	
Ward	
Village	
Latitude	(N), (S) _______°__________M
Longitude	(E), (W) _______°__________M
Elevation (meters)	
**Contact Information and household type**	
Telephone or mobile number	
Type of household (CODE 1)	
Name of data entry person (2)	

Introduction and Consent

[The objective is to interview a male adult and a female adult that identify themselves as household members that make most of the agricultural decisions. This is typically a husband and wife, however, if the principal decision-maker does not have a spouse but there is another adult in the household that makes agricultural decisions, these two people should be interviewed. There can also be cases where there is only one principal adult decision-maker in the household, in these cases, only interview him/her. Please do NOT replace a decision-maker for another person in the household because they are not available at the time of the interview.]

[Before starting the interview, read aloud the following paragraph and ensure that the respondents understand before asking for their consent.]

"Good morning/afternoon. We are coming from [PARTNER ORGANIZATION'S NAME]. We are conducting a survey to understand how farming decisions are made, what different agricultural practices you use, your household's access to information, extension services, and credit, and your participation in community groups. We would like to share some of this information widely in order that more people understand how food is grown and land managed in this region and the issues that you face regarding access to and use of agricultural information and credit.

Your household was randomly selected for this. Your name will not appear in any data that is made publicly available. The information you provide will be used purely for research purposes; your answers will not affect any benefits or subsidies you may receive now or in the future. Your participation in the survey is voluntary and you do not have to participate if you don't want to. You may withdraw from the study at any time and if there are questions that you would prefer not to answer then we respect your right not to answer them. You may ask questions at any time and if after the survey/interview you have any questions, you can contact [PERSON'S NAME] from [ORGANIZATION] at [PHONE NUMBER].

[IF INTERVIEWING A COUPLE]: This survey has two parts. In the first part, we would like to interview the principal couple in the household together; this part will take approximately one hour. In the second part, we would like to interview the two people separately; this part will take about one hour.
[IF INTERVIEWING ONLY ONE PERSON]: This interview will take about 2 hours.

Ask the adult male decision-maker:

Do you consent to provide information? Yes [ ] No [ ]

Ask the adult female decision-maker:

Do you consent to provide information? Yes [ ] No [ ]

Enumerator Name [ ]

Household ID [ ] Male Respondent ID [ ] Female Respondent ID [ ]

1. Household Composition *(Ask Couple together)*

	1.1	1.2	1.3	1.4	1.5	1.6	1.7	1.8	1.9					1.10
Member ID	Name of household members	What is the relationship to the household head?<br><br>1-Head<br>2-Spouse<br>3-Child<br>4-Step-child<br>5-Child-in-law<br>6-Grandchild<br>7-Parent<br>8-Parent-in-law<br>9-Sibling<br>10-Sibling-in-law<br>11-Other relative<br>12-Other, not relative<br>13-Employee	What year [NAME] born?	Sex<br><br>1-Male<br>2-Female	Marital status<br><br>1 – Married monogamous<br>2- Married-polygamous<br>3 – Separated/ Divorced<br>4- Widow/ Widower<br>5 – Single, never married	What level of education does [NAME] have?<br><br>0-None → Skip to 1.8<br>1-Primary<br>2-Secondary<br>3-Technical/ Vocational(not university)<br>4-University/ Diploma University/ degree<br>5-Graduate School<br>6-Other	What is the highest year completed at this level?<br><br>(Ask how the highest grade/level/year completed)	Which religion does [NAME] belong to?<br><br>1-Catholic<br>2-Protestant<br>3-Muslim<br>4-Adventist/ SDA<br>5-Pentecostal<br>6.Other<br>7.-None/Atheist	Does [NAME]…<br><br>1-Receives rents<br>2-Receives retirement pension<br>3- Receives subsidy or indemnification from government<br>4-Is a student<br>5-Is disabled or chronically ill<br>6-Is looking for work<br>7- Remittances<br>8- None of the above<br>9-Others (Specify)<br><br>[MARK ALL THAT APPLY]					What activity or occupation does [NAME] dedicate most of his/her time in a typical week? (Occupation is that in which they spend most of their time. Homemaker and/or student are valid occupations for the purposes of this survey.)
		**Code**	**Year**	**Code**	**Code**	**Code**	**Year**	**Code**	**Code**					
ID1														
ID2														
ID3														
ID4														
ID5														
ID6														
ID7														
ID8														
ID9														
ID10														
ID11														
ID12														

Household ID | | Male Respondent ID | | Female Respondent ID |

2. Labor Characteristics (Ask only for the 2 respondents)

	2.1	2.2	2.3	2.4	2.5
ID	In your job you are…?<br><br>**[READ THE ALTERNATIVES, CHOOSE ONLY ONE]**<br><br>1- Owner or partner<br>2- Self-employed<br>3- Unpaid family laborer<br>4- Government employee or worker<br>5- Private employee or worker<br>6- Day laborer<br>7- Domestic employee (maid, gardener, etc.)	Approximately how many hours do you work in a typical day in this occupation?	In a typical week, how many days do you work in this occupation?	How many months has [NAME] worked in this occupation in the last year? (If they answer all year; that's 12 months and less than one month should be 0)	How many years of experience does [NAME] have working in this activity/ occupation?
	**Position**	**Hours**	**Days**	**Months**	**Years**
ID1					
ID2					

	2.6	2.7	2.8	2.9	2.10	2.11	2.12	2.13	2.14
ID	Besides the activity just described, do you have a second occupation?<br><br>1-Yes<br><br>2-No → go to 2.12	What is your second occupation?	In your job you are…?<br><br>**[READ THE ALTERNATIVES, CHOOSE ONLY ONE]**<br><br>1- Owner or partner<br>2- Self-employed<br>3- Unpaid family laborer/homemaker<br>4- Government employee or worker<br>5- Private employee or worker<br>6- Day laborer<br>7- Domestic employee (maid, gardener, etc.)	Approximately, how many hours do you work in a typical day this (second) occupation?	In a typical week, how many days do you work in this (second) occupation?	How many months did you work in this occupation in the last year?	**[IF THE RESPONSE IN 2.1 or 2.7 IS "HOMEMAKER", DO NOT ASK THIS QUESTION, BUT WRITE 1 IN THIS QUESTION AND IN 2.14 WRITE THE NUMBER OF HOURS REPORTED IN 2.4.]**<br><br>Does [NAME] participate in the household chores (including cooking, fire wood collection, maintenance, childcare, carrying water etc.?<br><br>1- Yes<br>2- No → go to 3.1	Approximately, how many hours do you work in these activities in a typical day?	In a typical week, how many days do you do these types of activities?
	**CODE**	**OCCUPATION**	**CODE**	**HOURS**	**DAYS**	**MONTHS**	**CODE**	**HOURS**	**DAYS**
ID1									
ID2									

Household ID [ ] Male Respondent ID [ ] Female Respondent ID [ ]

3. Dwelling Characteristics and Well-being Indicators

3.1	3.2	3.3	3.4	3.5	3.6	3.7	3.8	3.9
How many years has your household lived here in this village continuously?	Does every household member have at least two sets of clothes?<br><br>1 – Yes<br>2 – No	Type of house/ dwelling?<br><br>**[Don't ask directly, mark according to observation]**<br><br>1-Independent House<br><br>3-Independent flat/ apartment<br>4-Sharing house/ flat/ apartment<br>5-Boys quarters<br><br>7- Hut<br><br>9- Other, specify	How many rooms does this household occupy?<br><br>**[Not including kitchen, bathrooms, garages, or rooms dedicated exclusively for business.]**	How many square meters is the dwelling?<br><br>**[Or use local unit/ measure and specify it below.]**	What is the main construction material used for the outside walls?<br><br>1- Thatch, straw<br>2- Mud and poles<br>3- Timber<br>4- Un-burnt bricks<br>5- Burnt bricks with mud<br>6- Burnt bricks with cement<br>7- Cement and blocks<br>8- Stone<br>9- Other, specify	What is the main construction material of the floor of the dwelling?<br><br>1- Earth<br>2-Earth and cow dung<br>3-Cement<br>4-Mosaic or tiles<br>5-Bricks<br>6-Stone<br>7-Wood<br>8-Other, specify	What is the main material used for the roof of the dwelling?<br><br>1 Thatch, straw<br>2 Mud<br>3 Wood<br>4 Iron sheets<br>5 Asbestos<br>6 Tiles<br>7 Tin<br>8 Concrete/ Cement<br>9 Tarpaulin<br>10 Other, specify	What type of toilet is mainly used by your household?<br><br>1. Covered pit latrine- private<br>2. Covered pit latrine- shared<br>3. VIP latrine- private<br>4. VIP latrine-shared<br>5. Uncovered pit latrine<br>6. Flush toilet- private<br>7. Flush toilet- shared<br>8. Bush<br>9. Other, specify
**YEARS**	**CODE**	**CODE**	**No. ROOMS**	**METERS SQUARED**	**CODE**	**CODE**	**CODE**	**CODE**

Household ID [ ] Male Respondent ID [ ] Female Respondent ID [ ]

Season	3.11<br>What is the principal source of lighting of the dwelling in [SEASON]?<br>1- Electricity from a Public Electric Company<br>2- Electricity from a Private Electric Plant<br>3- Electricity from a Generator<br>4- Solar Panels<br>5- Tin lamp<br>6- Candle<br>7- Rechargeable lamp<br>8- Gas<br>9- Liquid fuel (kerosene, petrol)<br>10- Battery powered source<br>11- Wood, sawdust or other natural material<br>12- None<br>13- Other, specify	3.12<br>What is the principal source of energy for cooking in [SEASON]?<br>1-Firewood<br>2-Dung<br>3. Crop residue<br>4. Kerosene<br>5. LP Gas<br>6. Charcoal<br>7. Solar<br>8. Electricity<br>9. -Other, specify	3.13<br>Do you use another energy source for cooking in [SEASON]?<br>1-Yes → Continue to 3.14<br>2-No → Next season	3.14<br>What is the other source?<br>1-Firewood<br>2-Dung<br>3. Crop residue<br>4. Kerosene<br>5. LP Gas<br>6. Charcoal<br>7. Solar<br>8. Electricity<br>9. Other, specify	3.15<br>**[If they respond 1 – firewood in 3.12 or 3.14, ask the following. If not, write "n/a" and continue with next season.]**<br>How much time do household members spend in a day collecting and carrying firewood?	3.16<br>Who is responsible for collecting and carrying the firewood?				
	**CODE**	**CODE**	**CODE**	**CODE**	**HOURS**	**IDs**				
Rainy season										
Dry season										

Season	3.17<br>Where does the household get its drinking water in [SEASON]?<br>1. Private connection to pipeline (tap) → Skip to 4.1<br>2. Public taps<br>3. Bore-hole<br>4. Protected well/ spring<br>5. Unprotected well/ spring<br>6. River, stream, lake, pond<br>7. Vendor/ Tanker truck → Skip to 4.1<br>8. Gravity flow scheme<br>9. Rain water → Skip to 4.1<br>10. Other, specify	3.18<br>How long does it take to collect the drinking water from this source?		3.19<br>Who collects and carries the water from this source?					3.20<br>How much time do household members take collecting and carrying water in a typical day?	3.21<br>How many days per week do household members collect and carry water?
	**CODE**	**Time in MINUTES**		**IDs**					**HOURS**	**DAYS**
		**To and From**	**Waiting Time**							
Rainy										
Dry										

Household ID ☐ Male Respondent ID ☐ Female Respondent ID ☐

4. Household Assets (Ask the couple together)

**Asset**	4.1<br><br>Does a household member own [ASSET]?<br><br>1-Yes→go to 4.2<br><br>2-No → next asset<br>Create code for joint ownership	4.2<br><br>Who are the owners of the [ASSET]?				4.3<br><br>Is there an ownership document?<br><br>1-Yes → go to 4.4<br>2-No → go to 4.5	4.4<br><br>Whose names appear on the document as owners?				4.5<br><br>How was the [ASSET] acquired?<br><br>1-Constructed<br>2-Purchased<br>3-Inherited<br>4-It was a donation<br>5 I started it<br>6-Other, specify
	**CODE**	**IDs**				**CODE**	**IDs**				**CODE**
House (Principal Residence)											
Other House											
Non-Agricultural Lot											
Business											

Household ID [ ] Male Respondent ID [ ] Female Respondent ID [ ]

	4.7<br><br>Does anyone in the household have [ASSET]?<br><br>1-Yes → go to 4.8<br>2-No → next asset	4.8<br><br>How many [ASSET] do you have (in the household)?	4.9<br><br>Who is/are the owner(s) of the [ASSET]?	What is the total value of assets? (Tshs)	Notes / Comments
	**CODE**	**NUMBER**	**IDs**		
Gas or Electric stove					
Refrigerator					
Washing machine					
Blender					
Radio					
Television					
DVD Player					
Mobile phone					
Furniture (i.e. Living Room and/or dining room set)					
Sewing machine					
Computer					
Fan					
Car / Pick-up truck					
Motorcycle					
Bicycle					
Generator (electricity)					
Water pump					
Water tank					
Solar Panel					
Wheel barrow					
Small agricultural tools					
Tractor					
Plough / Harvester / Other machinery implements					
Sprayer					
Dairy shed/Cattle Boma, Goats Pen / Chicken coup / etc.					
Mill					
Groundnut Sheller					
Other, specify					

Household ID [ ] Male Respondent ID [ ] Female Respondent ID [ ]

Section 5: Food Security

I would now like to ask you to describe a typical food year for your household. For each month say whether the food you consume is mainly from your own farm or from other sources. In addition, which months if any you tend to find you do not have enough food to eat for your family.

	JAN	FEB	MAR	APR	MAY	JUN	JUL	AUG	SEP	OCT	NOV	DEC
5.1. Source of food	[__]	[__]	[__]	[__]	[__]	[__]	[__]	[__]	[__]	[__]	[__]	[__]

FDSC *Codes for Q1: 1=Mainly from own farm, 2=Mainly from off farm (purchase/aid/other)*

5.2. Shortage /struggle to feed the family

[__]	[__]	[__]	[__]	[__]	[__]	[__]	[__]	[__]	[__]	[__]	[__]

HUNG *Codes for Q2: 1=Shortage, 0=No shortage*

Household ID | | Male Respondent ID | | Female Respondent ID | |

6. Credit and Insurance

6.1	6.2	6.3
Did anyone in your household ask for (or apply) for a loan or other type of credit in the last 12 months?<br><br>1-Yes<br>2-No → go to 6.4	Was the credit obtained?<br><br>1-Yes → go to 6.3<br>2-No → go to 6.4	Are you currently paying off the debt or is it already paid?<br><br>1-We're still paying<br>2-We already paid it off<br><br>>go to 6.4
**CODE**	**CODE**	**CODE**

[Fill in this table with all the debts that the household has CURRENTLY, if in 6.3 they responded "We're still paying" this debt should appear in the table. Be sure to ask for all current debts.]

	6.4	6.5			6.6	6.7	6.8	6.9	6.10		
**Debt Code**	Currently does any household member have a loan or owe someone money?<br><br>1-Yes → describe the debt and continue with 6.5<br><br>2-No → go to 6.11<br><br>**[If they respond 1, describe the debt.]**	Who in the household are responsible for the credit?			What is the source of the loan/ credit?<br><br>1-Family<br>2-Friend<br>3-Employer/Boss/Salary advance<br>4-Private Bank<br>5-Government owned bank (Agrarian bank)<br>6-Cooperative<br>7-Other private institution / NGO<br>8-Moneylender<br>9-Businees/store<br>10-Informal credit group<br>11-Credit card<br>12-Other, specify	In what year did you receive the loan?	What was the initial amount of the debt?	What was the purpose of the credit?	Who decided to ask for the credit?		
	**DESCRIPTION**	**IDs**			**CODE**	**YEAR**	**AMOUNT UGS**	**PURPOSE**	**IDs**		
D1											
D2											
D3											
D4											
D5											

Household ID		Male Respondent ID		Female Respondent ID	

Insurance Questions

Type of insurance	6.11<br><br>Does anyone in the household have [INSURANCE]?<br><br>1-Yes → go to 6.12<br>2-No → go to 6.13	6.12<br><br>Who has the insurance?						6.13<br><br>Why didn't you buy crop insurance?<br><br>1- Don't know about it<br>2- It's not available<br>3- It's not necessary<br>4-We don't have funds to buy it<br>5-Bad experiences in the past<br>6-Other, specify
	**CODE**	**IDs**						
Crop Insurance								
Livestock Insurance								
Health Insurance – public								
Health Insurance – private								
Funeral Insurance								
Life Insurance								
Home Owner Insurance								
Vehicle Insurance								

Household ID | Male Respondent ID | Female Respondent ID

7a. Sketch of the Farm (*to be done jointly by the couple*) -

Please sketch a quick map of all the plots that your household used throughout the past 12 months (OR ASK IF THEY HAVE A DRAWING OF THE FARM). This drawing should indicate a sketch of the farm, including intercropping and the seasonality when different crops appear in plots. It also includes ponds or grazing areas owned by the household. DO NOT FORGET TO ASK ABOUT PLOTS THAT ARE NOT OWNED BUT MAY BE RENTED, BORROWED, OR OTHERWISE USED BY THE HOUSEHOLD. This is to be done with both the male and female respondent together. Both enumerators—and their respondents—should agree on the number and order of the subplots that they have included.

Household ID ______ Male Respondent ID ______ Female Respondent ID ______

7b. Land Ownership and Production (Ask the couple together)

7.1<br><br>Plot number<br><br>**[Refer to sketch.]**<br><br>5.1.1 How many plots does the household have, including gardens?	7.2<br><br>Description or name of the plot	7.3<br><br>How many meters away from the household is the plot?	7.4<br><br>What is the area of the plot?<br><br>UNIT OF MEASURE:<br><br>1- Hectare<br>2-Square meters<br>3 - Acre		7.5<br><br>What is the tenure of the plot?<br><br>1-Owned and worked by household members → go to 7.6<br>2-Owned and rented out → go to 7.6<br>3-Owned and sharecropped out → go to 7.6<br>4-Owned and lent/ borrowed out → go to 7.6<br>5-Not owned, rented in → go to 7.11<br>6-Not owned, sharecropped in → go to 7.11<br>7-Not owned, borrowed → Skip to 7.11<br>8-Communal land<br>9-Other, specify	7.6<br><br>Who is/are the owner(s) of the plot?<br><br>USE HHID from HH rooster on page 2			7.7<br><br>How was the plot acquired?<br><br>1-Purchased<br>2-Customary /Inherited<br>3-It was a donation / gift<br>4-Communal<br>5-Other, specify	7.8<br><br>Do you have a title or other property document?<br><br>1-Yes → continue to 7.9<br><br>3-No →go to 7.11	7.9<br><br>What type of property document do you have?<br><br>1-Land Title<br>2 Lease document<br>3-Certificate of Customary Ownership<br>4.Sales Agreement<br>5. No Document (Skip to 7.11)6-Other, specify	7.10<br><br>Whose names are listed on the document as owners?	7.11<br><br>Who makes the major decisions regarding resource use and manages control over agricultural production on this plot?<br><br>[LIST ONLY ONE PERSON HERE.]
	**NAME / DESCRIPTION**	**METERS**	**#**	**UNIT**	**CODE**	**IDs**			**CODE**	**CODE**	**CODE**	**IDs**	**ID**
P1													
P2													
P3													
P4													
P5													
P6													
P7													
P8													
P9													
P10													

Household ID ☐ Male Respondent ID ☐ Female Respondent ID ☐

PLOT ID	7.11<br><br>Is this plot managed by household members?<br><br>1-Yes<br>2-No<br><br>[**Do not ask directly. If they respond 1, 5, 6, or 7 in 7.5write code 1, if not, go the next plot.**]	7.12<br><br>What did you cultivate on this plot in the first season 2014?<br><br>List Three major crops		7.13<br><br>Is this [CROP] intercropped?<br><br>1-Yes → With what other crop?<br><br>2-No → Continue to 7.14		7.14<br><br>How much area was cultivated with this crop(s)?<br><br>[**Verify that this is not greater than the total plot size in question 5.3**.]<br><br>UNITS OF MEASURE:<br>1-Hectare<br>2-Square meter<br>3-Acre		7.15<br><br>Do you irrigate this [CROP] on this subplot?<br><br>1-Yes → continue to 7.16<br>2-No → go to 7.19	7.16<br><br>What is the source of water for irrigation?<br><br>1-River, stream<br>2-Canal / channel<br>3-Constructed pond, reservoir, or pan<br>4-Natural lake or pond<br>5-Well, tubewell, or borehole<br>6-Sewerage water<br>7-Spring, natural<br>8-Other, specify<br><br>>continue with 7.17	7.17<br><br>What was the extraction method used for irrigation?<br><br>1 – Gravity<br>2 – Treadle/Foot pump<br>3 – Rower/Hand pump<br>4 – Diesel/Petrol pump<br>5 – Electric pump<br>6- Solar Water Pump<br>7- Wind Mills<br>8– Tractor pump<br>9– Bucket/hose<br>Other (specify)<br><br>>continue with 7.18	7.18<br><br>Do you pay for irrigation?<br><br>1. Yes, for the pump<br>2. Yes, for the water<br>3. Yes, for both<br>4. No
	**CODE**	**CROP(S)**			**CROP**	**#**	**UNIT**	**CODE**	**CODE**	**CODE**	
P1		P1.1									
		P1,2									
		P1.3									
P2		P2.1									
		P2.2									
		P2.3									
P3		P3.1									
		P3.2									
		P3.3									
P4		P4.1									
		P4.2									
		P4.3									
P5		P5.1									
		P5.2									
		P5.3									

Household ID [ ] Male Respondent ID [ ] Female Respondent ID [ ]

For the following tables please have each respondent (male and female) choose two plots that s/he manages or is most involved in the production activities (for a total of 4 sub-plots). List that sub-plot in the first column. It is important that you ensure that the sub-plot matches those listed in the above tables and in the sketch of the farm.

PLOTS	SUB-PLOT	7.19<br><br>Did you use fertilizers on this plot in the last year?<br><br>1-Yes, Farmyard manure<br>2- Yes, Compost<br>3-Yes, Inorganic fertilizer?<br>4- Yes, Other, specify<br>5- No → Skip to 7.20	7.19b<br><br>How much fertilizer did you use?	7.19c<br><br>Unit<br>1. kg<br>2. 50 kg bag<br>3 25 kg bag<br>4 other(specify)	7.19d<br><br>Price per unit if bought (Tzshs)	7.20<br><br>Did you use herbicides on this plot in the last year?<br><br>1-Yes<br>2-No → Skip to 7.21	7.20b<br><br>How much herbicide did you use?	7.20c<br><br>Unit<br>1. 1ltr/kg<br>2. 2ltr/kg<br>3. 5ltr/kg<br>4. 0.25 ltr/kg<br>5. 0.5 ltr/kg<br>6. other(specify)	7.20d<br><br>Price per unit if bought (Tzshs)	7.21<br><br>Did you use pesticides on this plot in the last year?<br><br>1-Yes<br>2-No → Skip to 7.22	7.21b<br><br>How much pesticide did you use?	7.21c<br><br>Unit<br>1. 1ltr/kg<br>2. 2ltr/kg<br>3. 5ltr/kg<br>4. 0.25 ltr/kg<br>5. 0.5 ltr/kg<br>6. other(specify)	7.21d<br><br>Price per unit if bought (Tzshs)
		**CODE**	**AMOUNT**	**CODE**	**USH**	**CODE**	**AMOUNT**	**UNIT**	**USH**	**CODE**	**AMOUNT**	**UNIT**	**USH**

Household ID [ ] Male Respondent ID [ ] Female Respondent ID [ ]

plotS	Sub-Plot	ACTIVITY<br><br>Land preparation<br>Planting<br>Weeding<br>Spraying<br>Harvesting	7.22<br><br>How many total adult person-days of work (including family and hired labor) were used on this plot for [ACTIVITY] in the first season of 2014?	7.23<br><br>How many person-days did female family members work in [ACTIVITY] on this plot in first season of 2014?	7.24<br><br>How many person-days did male family members work in [ACTIVITY] on this plot in the first season of 2014?	7.25<br><br>How many person-days were women hired to work in this [ACTIVITY] on this plot in the first season of 2014?	7.25b<br><br>What is the price per person-day of female labor?	7.26<br><br>How many person-days were men hired to work in this [ACTIVITY] on this plot in the first season of 2014?	7.26b<br><br>What is the price per person-day of male labor?
			**NUMBER**	**NUMBER**	**NUMBER**	**NUMBER**	**TZSH**	**NUMBER**	**TZSH**
		Land Preparation							
		Planting							
		Weeding							
		Spraying							
		Harvesting							
		Land Preparation							
		Planting							
		Weeding							
		Spraying							
		Harvesting							
		Land Preparation							
		Planting							
		Weeding							
		Spraying							
		Harvesting							

Household ID [ ] Male Respondent ID [ ] Female Respondent ID [ ]

SUB-PLOT	CROP	7.27a<br><br>How much was harvested in the second season of 2013?<br><br>UNITS OF MEASURE:<br><br>1-Basin<br>2-Kilogram<br>3-Stem / Racime (of bananas or plantains)<br>4-Sack / Bag<br>5-Tonnes<br>6-Tenga<br>7-No harvest (in the year)→next line<br><br>**[If they respond sack/bag or other measure not in kg, ask the weight of an average sack/bag in kgs.]**			7.28a<br><br>How much did you sell in the second season of 2013?<br><br>**[Verify that it's not greater than the amount harvested.]**<br><br>UNITS OF MEASURE:<br>1-Basin<br>2-Kilogram<br>3- Stem / Bunch (of bananas)<br>4-Sack / Bag<br>5-Tonnes<br>6-Tenga<br>7-Nothing sold → next line<br><br>**[If they respond sack/bag or other measure not in kg, ask the weight of an average sack/bag in kgs.]**			7.29a<br><br>What was the common sales price for the second season of 2013?<br><br>UNITS OF MEASUREMENT:<br><br>1-Basin<br>2-Kilogram<br>3-Stem/Racime<br>4-Sack/Bag<br>5-Tonnes<br>6- Tenga	
		**AMOUNT**	**UNIT**	**WEIGHT KG**	**AMOUNT**	**UNIT**	**Weight Kg**	**PRICE**	**UNIT**

Household ID [ ] Male Respondent ID [ ] Female Respondent ID [ ]

SUB-PLOT	CROP	7.27b<br><br>How much was harvested in the first season of 2014?<br><br>UNITS OF MEASURE:<br><br>1-Basin<br>2-Kilogram<br>3-Stem / Racime (of bananas or plantains)<br>4-Sack / Bag<br>5-Tonnes<br>6-Tenga<br>7-No harvest (in the year)→next line<br><br>**[If they respond sack/bag or other measure not in kg, ask the weight of an average sack/bag in kgs.]**			7.28b<br><br>How much did you sell in the first season of 2014?<br><br>**[Verify that it's not greater than the amount harvested.]**<br><br>UNITS OF MEASURE:<br>1-Basin<br>2-Kilogram<br>3- Stem / Bunch (of bananas)<br>4-Sack / Bag<br>5-Tonnes<br>6-Tenga<br>7-Nothing sold → next line<br><br>**[If they respond sack/bag or other measure not in kg, ask the weight of an average sack/bag in kgs.]**			7.29b<br><br>What was the common sales price for first season of 2014?<br><br>UNITS OF MEASUREMENT:<br><br>1-Basin<br>2-Kilogram<br>3-Stem/Racime<br>4-Sack/Bag<br>5-Tonnes<br>6- Tenga	
		**AMOUNT**	**UNIT**	**WEIGHT KG**	**AMOUNT**	**UNIT**	**Weight Kg**	**PRICE**	**UNIT**

Household ID ☐ Male Respondent ID ☐ Female Respondent ID ☐

LIVESTOCK OWNERSHIP

Type of livestock	Age of animal (months)	Breed<br>1= Exotic<br>2= Indigenous	Sex of the animal	During the last 12 months, has any member of your household raised or owned […]?<br>1=Yes<br>2=No (>> NEXT ANIMAL)	How many of the livestock are: a) owned by your household now (present at your farm or away)<br>b) kept but not owned by your household now?	If you would sell one of your own […] today, how much would you receive from the sale?	How many did you own exactly 12 months ago (present or away)?	During the last 12 months how many were received as gifts?	During the last 12 months how many were lost or died?	During the last 12 months how many were given as gifts?	During the last 12 months how many were bought?	During the last 12 months how many were slaughtered?

Household ID | Male Respondent ID | Female Respondent ID

LIVESTOCK EXPENDITURE

Type of expenditure<br><br>Codes:<br>1=Hired labour for<br>2= Livestock feed<br>3= veterinary services/medicine<br>4=other expense (specify)	Did you spend any on [……]<br>1=Yes<br>2=No	Cash value (if it is in kind give estimated cash value)
**CODE**		

Household ID ☐ Male Respondent ID ☐ Female Respondent ID ☐

LIVESTOCK PRODUCTS AND INCOME

Livestock product<br><br>(1) Cow Milk<br><br>(2) Goat Milk<br><br>(3) Sour Milk<br><br>(4) Ghee<br><br>(5) Eggs<br><br>(6)Honey<br><br>(7) Beef<br><br>(8) Chicken<br><br>(9) Goat Meat<br><br>(10) Mutton<br><br>(11) Pork<br><br>(12) Other Meat<br><br>(13) Hides and Skin<br><br>(14) Blood<br><br>(15) Other (Specify)	Number of Production months in the last 12 months	Average production per month (during production months)				What was the average consumption per month (during production months)	Who controls the revenue from this product?<br><br>Record Person ID (Up to 2 IDs		Where/to whom do you mainly sell your products?<br><br>1=Government/LC<br><br>2=Private trader in local market/village in district market<br><br>3=Private trader in district market<br><br>4Consumer at market<br><br>5=Neighbor/Relative<br><br>6=Other (Specify)	
		Qty	Unit of Prod<br><br>1=Kgs<br><br>2=Litres<br><br>3=Trays<br><br>4=Numbers<br><br>6=Other (Specify)	Qty	Total Value in Tshs					

THIS POINT, INTERVIEW THE COUPLE SEPARATELY.

Household ID ______ Male Respondent ID ______ Female Respondent ID ______

8. Decision Making (Ask each decision maker separately)

Fill in the sub-plots used in the last section and ask these decision-making questions about those sub-plots.

Sub-Plots	8.1.1<br><br>Who decides what crops to plant on this sub-plot?	8.1.2<br><br>Who decides how to prepare the land for planting?	8.1.3<br><br>Who decides if you will use fertilizers (including organic)?<br><br>If you do not use fertilizers, who decides not to use them?	8.1.4<br><br>Who decides what inputs you use?	8.1.5<br><br>Who decides to hire labor (or not hire labor)?	8.1.6<br><br>Who decides how to control weeds (manual weeding, use of herbicides, etc.)?	8.1.7<br><br>Who decides when to begin harvesting?	8.1.8<br><br>Who decides how much of the harvest to sell?	8.1.9<br><br>Who decides how to use the income from other sale of the crop?
	**IDs / CODE**	**IDs / CODE**	**IDs / CODE**	**IDs / CODE**	**IDs / CODE**	**IDs / CODE**	**IDs / CODE**	**IDs / CODE**	**IDs / CODE**

Household ID

Male Respondent ID

Female Respondent ID

Module 8.2

**8.2.1** **Currently how many [ANIMAL] does the household have?** **[Write 0 if the household does not have any of this animal]**		8.2.2 Who in the household are the owners of the [ANIMAL]?			8.2.3 Who is usually in charge of giving water to[ANIMAL] usually?			8.2.4 Who decides what to feed the [ANIMAL] usually?			8.2.5 Who is usually in charge of the [ANIMAL] health?			8.2.6 Who decides where the [ANIMAL] will graze usually?			8.2.7 Who decides about the reproduction of the [ANIMAL] usually?			8.2.8 Who decides when to slaughter an [ANIMAL] usually?		
		**IDs**			**IDs/COD**			**IDs/COD**			**IDs/COD**			**IDs/COD**			**IDs/COD**			**IDs/COD**		
Cattle																						
Milk Cows																						
Oxen																						
Goats																						
Sheep																						
Rabbits/ Guinea pigs																						
Chicken																						
Other Poultry																						
Pigs																						
Bees																						
Fish																						
Donkeys																						

Household ID ______ Male Respondent ID ______ Female Respondent ID ______

Currently how many [ANIMAL] does the household have? [Write 0 if the household does not have any of this animal]	8.2.9 Who is in charge of [ANIMAL PRODUCT] usually? (e,g eggs, milk, meat, manure)			8.2.10 Did you sell any of the [ANIMAL PRO-DUCT] in the last 12 months? 1-Yes →Go to 8.2.11 2-No→Go to 8.2.13	8.2.11 Who normally decides how much to sell of [ANIMAL PRO-DUCT]?			8.2.12 Who normally decides how to use the income from the sale of [ANIMAL PRODUCT]?			8.2.13 Did you sell [ANIMAL] in the last 12 months? 1-Yes 2-No→Skip to the next animal	8.2.14 Who decided to sell the [ANIMAL]?			8.2.15 Who decided how to use the income obtained through the sale of the [ANIMAL]?		
	**IDs/COD**			**CATEGORY**	**IDs/COD**			**IDs/COD**			**CATEGORY**	**IDs/COD**			**IDs/COD**		
Cattle																	
Milk Cows																	
Oxen																	
Goats																	
Sheep																	
Rabbits/Guinea pigs																	
Chicken																	
Other Poultry																	
Pigs																	
Bees																	
Fish																	
Donkeys																	

Household ID | Male Respondent ID | Female Respondent ID

Module 8.3

**Activity**		8.3.1<br><br>In your household in the last five years have you [ACTIVITY]?<br><br>1-Yes → Please describe and continue to 8.25<br>2-No → Skip to the next activity		8.3.2<br><br>Who decided to do it?			
		**CATEGORY**	**DESCRIPTION**	**IDs / CÓDE**			
**A**	Made important agricultural investments (machinery, infrastructure, irrigation)						
**B**	Bought or sold land						
**C**	Rented land						
**D**	Purchased major household ítems (appliances, furniture, etc.)						

**Expenses**		8.3.3<br><br>Who decides about the Budget for [expenses]?			
		**IDs / CODE**			
**E**	Food costs for the household				
**F**	Expenses for the children (clothing, school supplies, uniforms)				

Household ID [ ] Male Respondent ID [ ] Female Respondent ID [ ]

8.3.4<br><br>In the last one year have you worked off-farm?<br><br>**[It is not necessary to ask directly, you can verify from the labor module (module 2)]**<br><br>1-Yes, in agricultural activities<br>2-Yes, in nonagricultural activities<br>3-Yes, in both agricultural nonagricultural activities<br>4-No → Skip to 8.3.7	8.3.5<br><br>Did you make these decisions…?<br><br>1- By yourself<br>2- Together with<br>3- With permission from<br>4- Other person took the decision	8.3.6<br><br>About the income that you received, how did you decide to spend it?<br><br>1- I decide myself about what I earn<br>2- I decide by myself about a part of it, but for another part I discuss with **(WRITE ID OF OTHER PERSON)**<br>3- I decide what to do with what I earn, jointly with: **(WRITE ID OF OTHER PERSON)**<br>4- I do not decide, another person does **(WRITE ID OF OTHER PERSON)**		8.3.7<br><br>Has anyone in your household received remittances in the last year?<br><br>1- Yes, who?<br>2- No-Skip to 8.3.11		8.3.8<br><br>How often do you receive this remittance?<br><br>1- Daily<br>2- Every 2 weeks<br>3- Monthly<br>4- Every 2 months<br>5- Every 3 months<br>6- Every 6 months<br>7- Annually<br>8- Other, specify	8.3.9<br><br>How much do remittances contribute to household income?<br><br>1-Very little<br>2-Little<br>3-Half<br>4-More than half<br>5-The majority	8.3.10<br><br>Whom do you receive this remittance from?
**CODE**	**CODE**	**CODE**	**ID**	**CODE**	**ID**	**CODE**	**CODE**	**WRITE IN RESPONSE**

8.3.11<br><br>Who in the household earns the most money from off-farm activities?<br><br>**(Write the ID code of one person unless 2 or more people earn equally in off-farm activities.)**			8.3.12<br><br>From these off-farm activities, who contributes the most to household expenses?			8.3.13<br><br>Keeping in mind all of the sources of income, including from your farm and off-farm activities, which is the **most** important for the maintenance/subsistence of the household?<br>1-Agriculture (from the farm)<br>2-Off-farm work (day labor/contract work)<br>3-Business<br>4-Other, specify	8.3.14<br><br>Of all the foods that are consumed in the household, what proportion is purchased?<br><br>**(For pre-testing see which works best "purchased" or "produced on the farm".)**<br><br>1-Very little<br>2-Little<br>3-Half<br>4-More than half<br>5-The majority
**IDs**			**IDs**			**Category**	**Category**

Household ID | | Male Respondent ID | | Female Respondent ID |

9. Group Memberships *(Ask each decision-maker separately)*

		9.1	9.2	9.3	9.4	9.5	9.6
**Categories of Groups**		Is there a [GROUP] in your community<br><br>1Yes<br>2-No-->skip to the next group	Are you a member of this group?<br><br>1-Yes<br>2-No --> skip to next group	Do you usually attend meetings of this group?<br><br>1Yes<br>2-No	Are you, or have you been in the last 5 years, elected to a leadership position in this group?<br><br>1-Yes<br>2-No→ Skip to next group	Do you feel comfortable speaking up in this group to give your opinion or offer suggestions?<br><br>1- No, absolutely not<br>2- Yes, with difficulty<br>3- Yes, I am very comfortable	How much influence do you have in making decisions in this [GROUP]?<br><br>1 –No influence<br>2 –Influence in some decisions<br>3 –Influence in the majority of decisions
			**Category**	**Category**	**Category**	**Category**	**Category**
**A**	Agricultural Producers Group (including Marketing Groups)						
**B**	Livestock Producer Groups (Including marketing Groups)						
**C**	Fishery Producers Groups (Including marketing Groups)						
**D**	Water User Groups						
**E**	Watershed Management Groups						
**F**	Forest User Groups						
**G**	Tree Nursery Groups/Reforestation Groups						
**H**	Credit, Microfinance, or Savings Groups						
**I**	Funeral/Burial or Insurance Group						
**J**	Income and marketing groups (nonagricultural)						
**K**	Civic or charitable groups (improving the community or helping others)						
**L**	Local Committees (neighborhood/village)						
**M**	Religious Groups						
**N**	Youth Group						
**O**	Other; specify						

Household ID [ ] Male Respondent ID [ ] Female Respondent ID [ ]

10. Practices (Ask each decision-maker separately)

Please remind the respondents that these answers should be from their point of view (whether the person knows and does the practice—not the household).

	10.1	10.2	10.3	10.4	10.5	10.6			10.7	10.8
**Practice**	Do you know about or have you heard about PRACTICE]?)<br><br>1-Yes→ Continue to 10.2<br><br>2-No→ Next practice	What is your primary / principal source of information regarding [PRACTICE]?	In the last 12 months, have you used [PRÁCTICE] on any of your plots?<br><br>1-Yes → Continue to 10.4<br><br>2-No → Skip to 10.7	On which sub-plots did you use this practice?<br><br>Indicate as: 1.1 (plot 1, sub-plot 1).	When did you begin to use this practice?	Who decided to start using this practice?<br><br>Skip to Next practice.			Did you use this [PRÁCTICE] previously on your plots?<br><br>1-Yes → Go to 10.8<br><br>2-No→ next practice	Why did you stop using this [PRÁCTICE]?<br><br>7> Next practice
	**CATEGORY**		**CATEGORY**	**Sub-plots**	**Year**	**IDs/CÓDE**				
Agroforestry										
Live fences or wind breaks –										
Terracing										
Drainage Ditches/channels										
Minimum Tillage										
Farmyard manure compositing for application on crop										
Compost / Vermiculture										
Mulching										
Appropriate use of fertilizers according to soil test										
Cover Cropping										
Green Manure										
Intercropping										
Use of Improved Varieties										
Integrated Pest Management										
Zero grazing										
No Burning										
Fallow										
Stone lining										
Contour ploughing										

Household ID ______ Male Respondent ID ______ Female Respondent ID ______

(Continuation of practices table.)

	10.11	10.12	10.13	10.14	10.15	10.16			10.17	10.18
**Practice**	Do you know about or have you heard about PRACTICE]?)<br><br>1-Yes→ Continue to 10.12<br><br>2-No→ Next practice	What is your primary / principal source of information regarding [PRACTICE]?	In the last 12 months, have you used [PRÁCTICE] on any of your plots?<br><br>1-Yes → Continue to 10.14<br><br>2-No → Skip to 10.17	On which sub-plots did you use this practice?	When did you begin to use this practice?	Who decided to start using this practice?			Did you use this [PRÁCTICE] previously on your plots?<br><br>1-Yes → Skip to 10.18<br><br>2-No→ next practice	Why did you stop using this [PRÁCTICE]?<br><br>> Next practice
	**CATEGORY**	**CODE**	**CATEGORY**	**Sub-plots**	**Year**	**IDs/CÓDE**				
Improved livestock breeds										
Improved Pasture										
Pasture Rotation										
Irrigation										
Water storage for irrigation										
Improved processing of grains										
Use of fuel-efficient or improved stoves										
Seed selection or seed preservation for planting in future seasons										
Other, specify										

Household ID [ ] Male Respondent ID [ ] Female Respondent ID [ ]

11. Sources and types of information *(Ask each decision-maker separately.)*

Source	11.1<br><br>Did you receive agricultural or climatic information from this [SOURCE]?<br><br>1 - Yes<br>2 - No → next source<br><br>**CATEGORY**
Government Extension Workers	
Farmer or Community Organizations	
NGOs	
Agriculture Service Providers (Selling Seeds, fertilizers, etc)	
Agriculture Fairs	
Demonstration/Field Days	
Family Members	
Neighbors	
Radio	
TV	
Newspapers/Magazines	
Text Messages	
Internet (through computer/cellphone)	
Nature (natural indicators, own knowledge, traditional knowledge)	
Other, specify	

Household ID | Male Respondent ID | Female Respondent ID

Types of Information	11.2<br>Have you received this [TYPE] of information?<br><br>1-Yes→Continue to 11.3<br><br>2-No→ Go to next type of information	11.3<br>What was the source of this information?	11.4<br>Were you able to use this information for your agricultural activities?<br><br>1- Yes<br>2- No
	**CATEGORY**	**SOURCE**	**CATEGORY**
Short term rain Forecast (daily, weekly, monthly)			
Medium and Long Term Rain Forecast (drought, 2-3 months)			
Information about crop production and management			
Post-harvest management			
Information about the production and management of livestock			

Household ID | Male Respondent ID | Female Respondent ID

12. Climatic shocks *(Ask each decision-maker separately)*

12.1	12.2	12.3	12.4	12.5	12.6	12.7	12.8	12.9
What natural climate shocks have affected your household (income or agriculture) during the last five years?<br><br>0- None → Go to section 13<br>1- Floods<br>2- droughts<br>3- storms/ strong winds<br>4- irregular rain<br>5- Frost/Cold Temperatures<br>6- Heat Waves<br>7- Fires<br>8- Land Slides<br>9-Earthquakes<br>10- Other, specify	When was the last climatic shock?<br><br>**[Year, in the last five years]**	The shock impacted:<br><br>1- Only my household<br>2- Some households in the community<br>3- The majority of the households in the community<br>4-Many households in the community.	What were the primary damages of the shock (to the household)?<br><br>(Name up to three damages)	What immediate actions did members of your household take to cope with the damages?<br><br>(Name up to three actions)	Who decided to take the action?	**[Verify if the household sold assets, borrowed money or used savings for this shock]**<br><br>**If they do not mention this in action 12.5, please ask.**<br><br>1-Yes, sold assets→ go to 12.8<br><br>2-Yes, used savings or borrowed→ go to 12.9<br><br>3-No→next event	What assets did you sell?	Who was the owner/solicitor/ borrower?
**CODE**	**YEAR**	**CATEGORY**	**DAMAGES**	**ACTIONS**	**IDs/CODES**	**CATEGORY**		**IDs/CODES**
SHOCK 1								
SHOCK 2								
SHOCK 3								
SHOCK 4								

Household ID [ ] Male Respondent ID [ ] Female Respondent ID [ ]

13. Perceptions of climate change and associated risks *(Ask each decision-maker separately.)*

13.1 Comparing the climate and/or weather in your youth and that of today, have you noticed any changes in the climate?

1-Yes → Continue to 13.2
2-No → Skip to 13.3A (next column)

CATEGORY	

13.2 What changes have you noticed?

13.3 How likely do you think it is that there will be climate changes in the futures?

1-Exteremly unlikely → Skip to Section 14
2-Somewhat likely
3-Likely
4-Very likely

CATEGORY	

13. 4 How much of an impact do you think that these changes will have on your household's livelihood?

1-No impact
2-Small impact
3-Medium impact
4-Strong Impact

CATEGORY	

13.5 What are you most worried about in regards to climate (or weather)?

Go to Section 14.

13.3A How likely do you think it is that there will be climate changes in the futures?

1-Exteremly unlikely → Go to Section 14
2-Somewhat likely
3-Likely
4-Very likely

CATEGORY	

13. 4A How much of an impact do you think that these changes will have on your household's livelihood?

1-No impact
2-Small impact
3-Medium impact
4-Strong Impact

CATEGORY	

13.5A What are you most worried about in regards to climate (or weather)?

Go to Section 14.

Household ID [ ] Male Respondent ID [ ] Female Respondent ID [ ]

14. Adaptation *(Ask each decision-maker separately.)*

14.1	14.2	14.3			14.4	14.5	14.6	14.7	14.8
Have you made any changes to protect yourself, your family, or your community (from climate changes)?<br><br>This can include any changes in agriculture, livestock, or livelihood strategies.<br><br>1-Yes→ skip to 14.2<br>2-No → section 15	What changes have you made?<br><br>Name the most important (up to 5)	Who in the household decided to make these changes?			Do you plan to make any (additional) changes to protect against changes in climate over the next 5 years?<br><br>1- Yes>>14.5<br>2- No<br>>> 14.6<br>3- Don't know/ not sure>>14.6	If yes, what changes do you plan to make?	Are there any changes you would like to make but are not able to in the near future?<br><br>1-Yes→ Go to 14.7<br>2-No→Section 15<br>3-Don't Know/Not Sure → section 15	What changes would you like to make?	Why is it not possible to make these changes?<br><br>> Skip to section 15
**CODE**		**IDs / CÖDE**			**CATEGORY**		**CATEGORY**		
	Change 1:					Change 1:		Change 1:	
	Change 2:					Change 2:		Change 2:	
	Change 3:					Change 3:		Change 3:	
	Change 4:					Change 4:		Change 4:	
	Change 5:					Change 5:		Change 5:	

Household ID [ ] Male Respondent ID [ ] Female Respondent ID [ ]

15. Gender Roles & Personal Values

Please indicate the level of agreement with these questions:

		**1 - Strongly Disagree**	**2 - Somewhat disagree**	**3 – Neither Agree nor disagree**	**4 - Somewhat Agree**	**5 - Strongly agree**
15.1	Women and men have the same right to make their own decisions.					
15.2	A good wife should obey her husband even if she does not agree with him.					
15.3	A woman has the same capacity to earn money as a man.					
15.4	Women have the right to live a life free of violence.					
15.5	The man should take responsibility for all of the family expenses					
15.6	It's the woman's obligation to have sexual relations with her husband even if she does not want to.					
15.7	The woman should be free to decide if she wants to work outside the home.					
15.8	Men should have more time to rest and relax than women.					
15.9	Women have the right to defend themselves and to report to the authorities any mistreatment or aggression.					
15.10	Childcare should be shared between spouses.					
15.11	Women and men should have the same freedom for professional development.					
	In your opinion, a husband is justified in hitting his wife in the following situations:					
15.12	If she goes out without telling him?					
15.13	If she neglects the children?					
15.14	If she argues with him.					
15.15	If she refuses to have sex with him?					
15.16	If she disobeys her husband.					
15.17	If she burns the food.					
15.18	If she is having an affair.					

15.19 Have you received some training on these issues?

1-Yes
2-No

[ ]

Household ID [ ] Male Respondent ID [ ] Female Respondent ID [ ]

We are interested in knowing which life-guiding principles or values you have that guide your choices and decisions. With that in mind, please rate the following statements based on your own personal values.

	Statement	1 - Strongly Disagree	2 - Somewhat disagree	3 – Neither Agree nor disagree	4 - Somewhat Agree	5 - Strongly agree
15.20	I actively seek out advice about agricultural practices for my farm.					
15.21	If spouses (males and females) make household agricultural decisions together, their livelihood will improve (agricultural productivity, food security, income etc).					
15.22	It is important to challenge oneself and to learn and try new things.					
15.23	I highly value new agricultural information, technology, and weather information.					
15.24	Members of the community should work together to improve the community (maintaining common areas, infrastructure, etc.).					
15.25	I am often one of the first people in my community to try new practices on my farm.					
15.26	When making agricultural decisions, I am most concerned about generating income.					
15.27	I am willing to accept agricultural advice from outside sources.					
15.28	I make my own agricultural decisions without worrying about what other people say.					
15.29	My community is welcoming to new agricultural ideas and practices.					
15.30	Co-operation with others usually works.					
15.31	Being a farmer is an important part of my identity.					
15.32	When making agricultural decisions, I am most (very) concerned about food security.					
15.33	I am capable of improving my life and the lives of members of my household.					
15.34	I trust members of my community to help me in times of need.					
15.35	I trust my family to help me in times of need.					
15.36	It is important to me to be able to pass my farm/land on to my children.					
15.37	I feel a very strong connection to the land that I farm.					
15.38	Men and women should have equal roles in agricultural decision-making					

Many thanks!
